# Expanding HIV-1 subtype B transmission networks among men who have sex with men in Poland

**DOI:** 10.1371/journal.pone.0172473

**Published:** 2017-02-24

**Authors:** Miłosz Parczewski, Magdalena Leszczyszyn-Pynka, Magdalena Witak-Jędra, Bartosz Szetela, Jacek Gąsiorowski, Brygida Knysz, Monika Bociąga-Jasik, Paweł Skwara, Anna Grzeszczuk, Maria Jankowska, Grażyna Barałkiewicz, Iwona Mozer-Lisewska, Władysław Łojewski, Katarzyna Kozieł, Edyta Grąbczewska, Elżbieta Jabłonowska, Anna Urbańska

**Affiliations:** 1 Department of Infectious, Tropical Diseases and Immune Deficiency, Pomeranian Medical University in Szczecin, Szczecin, Poland; 2 Department of Infectious Diseases, Hepatology and Acquired Immune Deficiencies, Wrocław Medical University, Wrocław, Poland; 3 Department of Infectious Diseases, Jagiellonian University Medical College, Kraków, Poland; 4 Department of Infectious Diseases and Hepatology, Medical University of Bialystok, Białystok, Poland; 5 Department of Infectious Diseases, Medical University in Gdańsk, Gdańsk, Poland; 6 Department of Infectious Diseases, J. Strus Hospital, Poznań, Poland; 7 Department of Infectious Diseases, Poznań University of Medical Sciences, Poznań, Poland; 8 Department of Infectious Diseases, Regional Hospital in Zielona Gora, Zielona Góra, Poland; 9 Department of Infectious Diseases and Hepatology Nicolaus Copernicus University, Collegium Medicum in Bydgoszcz, Bydgoszcz, Poland; 10 Department of Infectious Diseases and Hepatology, Medical University of Łódź, Łódź, Poland; Fudan University, CHINA

## Abstract

**Introduction:**

Reconstruction of HIV transmission links allows to trace the spread and dynamics of infection and guide epidemiological interventions. The aim of this study was to characterize transmission networks among subtype B infected patients from Poland.

**Material and methods:**

Maximum likelihood phylogenenetic trees were inferred from 966 HIV-1 subtype B protease/reverse transcriptase sequences from patients followed up in nine Polish HIV centers. Monophyletic clusters were identified using 3% within-cluster distance and 0.9 bootstrap values. Interregional links for the clusters were investigated and time from infection to onward transmission estimated using Bayesian dated MCMC phylogeny.

**Results:**

Three hundred twenty one (33.2%) sequences formed 109 clusters, including ten clusters of ≥5 sequences (n = 81, 8.4%). Transmission networks were more common among MSM (234 sequences, 68.6%) compared to other infection routes (injection drug use: 28 (8.2%) and heterosexual transmissions: 59 (17.3%) cases, respectively [OR:3.5 (95%CI:2.6–4.6),p<0.001]. Frequency of clustering increased from 26.92% in 2009 to 50.6% in 2014 [OR:1.18 (95%CI:1.06–1.31),p = 0.0026; slope +2.8%/year] with median time to onward transmission within clusters of 1.38 (IQR:0.59–2.52) years. In multivariate models clustering was associated with both MSM transmission route [OR:2.24 (95%CI:1.38–3.65),p<0.001] and asymptomatic stage of HIV infection [OR:1.93 (95%CI:1.4–2.64),p<0.0001]. Additionally, interregional networks were linked to MSM transmissions [OR:4.7 (95%CI:2.55–8.96),p<0.001].

**Conclusions:**

Reconstruction of the HIV-1 subtype B transmission patterns reveals increasing degree of clustering and existence of interregional networks among Polish MSM. Dated phylogeny confirms the association between onward transmission and recent infections. High transmission dynamics among Polish MSM emphasizes the necessity for active testing and early treatment in this group.

## Introduction

Scale up of antiretroviral treatment (ART), mother-to-child transmission reduction programs and earlier introduction of ART have resulted in decreased transmission of new HIV infections worldwide, especially in Africa and Asia [1,2]. However, in the recent years increase in the number of the new cases has been observed in high and middle income European countries.

This increase was associated with injection drug use (IDU) in Eastern Europe including countries such as Russia, Ukraine and Belarus and upsurge of new infections among men-who-have-sex-with-men (MSM) in West European countries [3,4]. HIV-1 epidemics in Poland was historically associated with IDU, which was the predominant route of infection until 2005 [5]. However, in recent years a shift in the dominant transmission route was observed with a 14-fold increase in the infection frequency among MSM—from 2.5 new diagnoses per million of male inhabitants in 2000 to 33.8 in 2011 and beyond [6] as well as a 90% decline in the IDU related transmissions [7]. Currently more than 70% of new diagnoses are amongst MSM aged 25–34 years [8]. Also, recent models have indicated a high proportion (68.3%, 95%CI 53.9–76.1%) of undiagnosed HIV among MSM, which is related to low testing rates in Poland and lack of uniform preventive strategy focused on this group, despite higher testing rates associated with MSM exposure [9,10].

It has been reported that new infections were associated with inconsistent condom use and concentrated in the large cities, however, factors driving this epidemics in Poland remain largely understudied [6]. Across HIV infected populations subtype B remained stably the most common HIV-1 variant (95.8–86.1%) [11,12] and associated with male gender, MSM transmissions, higher CD4 lymphocyte counts and lower HIV plasma viremia at care entry [13,14].

Phylogenetics provides an insight into the HIV network structure including its transmission dynamics [15]. Use of dated sequence data, coupled with demographic and clinical information allows to model characteristics of the epidemics including population-based patterns of HIV transmission [16]. Dated sequence analyses from cohorts with known and unknown infection time, showed that early infection was associated with onward transmissions in 25–50% of cases [17,18,19,20]. For above-mentioned purposes HIV-1 partial *pol* sequences obtained from routine genotyping assays have been successfully used. Characterizing populations forming transmission networks allows to target interventions to the ones at risk. Using phylogeny relationship between various viral strains may be established with identification of closely interrelated subsets—phylogenetic clusters [19]. Typically, a cluster contains all taxa—in case of HIV analyses patient derived sequences—descending from the similar lineage or ancestry [21]. Using these clusters chronology of the spread viral clades spread may be visualized. Typically clusters are defined by the genetic discane between analyzed sequences and/or statistical support (bootstrap) within the inferred tree [22,23,24]. In HIV medicine clustering dynamics may reflect changing patient mobility and allows to assess the efficacy of ART as prevention strategy [19,25,26].

Having previously identified common clustering among MSM [13], in this study we wished to investigate interregional links and time-trends associated with transmission networks for the HIV-1 subtype B. The aim of this study was to provide insight, based on the phylogenetic data, into the characteristics and trends in the dynamics of HIV transmission in Poland. We also aimed to investigate network patterns on the national level to guide HIV prevention.

## Materials and methods

### Study population and sequencing

For this study 966 HIV-1 subtype B samples collected from patients with defined transmission route for the purpose of genotypic drug resistance analyses were used. Patients were linked to care in 9 of 17 Polish HIV treatment centres (Białystok, Bydgoszcz, Gdańsk, Łódź, Kraków, Poznan, Szczecin, Wroclaw, Zielona Gora). Sequencing was performed at the Clinical Laboratory at the Department of Infectious, Tropical Diseases and Acquired Immune Deficiency, Pomeranian Medical University in Szczecin, Poland.

The samples included sequences collected both from treatment-experienced (n = 176, 18.21%) and antiretroviral-naive (n = 790, 81.87%) patients diagnosed in Poland from 1989 to 2014, prior to the introduction of the antiretroviral treatment. Data for subtype B treatment-naive patients were partially collected as a part of the Northern-Poland Resistance Transmission Tracking for HIV-1 (NoRTTH) project funded by the Polish AIDS Society, and the resistance data were previously published elsewhere [13]. For the molecular clock analyses sequence sampling dates were used. For individuals with multiple sequences available, the earliest sequence after the positive confirmation HIV test, was included.

The analysed data included: gender, CD4 lymphocyte count and HIV viral load at care entry, CD4 lymphocyte nadir, age at diagnosis (the age of first positive confirmation test), HCV co-infection (defined as presence of anti-HCV antibody or HCV-RNA), transmission route (self-defined by the patient) and CDC clinical stage according to the 1993 classification system for HIV infection [27] at care entry. As seroconversion time point was often unavailable in the source documentation data for the acute HIV infections were not collected—date of infection was based on the date of the first positive and confirmed HIV test.

The study protocol was approved by the institutional review board named Bioethical Committee of Pomeranian Medical University in Szczecin (Komisja Bioetyczna Pomorskiego Uniwersytetu Medycznego w Szczecinie), Poland (approval number KB-0012/08/12). Research was conducted in accordance with the Declaration of Helsinki. As the patient data were coded and anonymous, while all the procedures, including genotyping, are the part of the medical care on the people living with HIV, no separate written consent was obtained but physicians informed all subjects on the planned research and checked for verbal non-opposition from their patients.

### Sequencing

HIV RNA extraction, as well as reverse transcriptase and protease genotyping, were performed using a genotyping assay (Viroseq 2.8, Abbott Molecular, Abbott Park, Illinois, USA) according to the manufacturer’s protocol. Protease/reverse transcriptase sequences obtained were 1302 b.p. long; location from the start of HXB2 genome: positions 2253 to 3525. Identification of subtype B was performed based on the partial *pol* sequences using two methods: on-line software (REGA genotyping 2.0 tool; http://bioafrica.mrc.ac.za/rega-genotype/html/subtypinghiv.html) and phylogenetic analyses using maximum likelihood (ML) method under the GTR+γ model with approximate likelihood ratio test (aLRT) (online PHYMLv.3.0-http://www.atgc-montpellier.fr/phyml) [28]. For reference, sequences with known subtype from the 2014 version of the HIV sequence compendium (Los Alamos National Laboratory) were used, supplemented with CRFs from the HIV sequence database (http://www.hiv.lanl.gov/components/sequence/HIV/search/search.html). All sutype B sequences included in the analysis were assessed for recombination using two methods: a simplot bootscan (2-parameter Kimura model, window size 200 b.p., 20.p. step) [29] and jumping-profile hidden Markov model (jpHMM) [30]. Recombinant sequences were removed from the final dataset. Thirty-seven codon sites associated with protease and reverse transcriptase resistance mutations were manually deleted from the sequence alignments. The final alignment was 1191 b.p. long.

### Identification of clustering

First, clusters were identified using the Maximum Likelihood (ML) method with the NNI-SPR sub-tree algorithm under the GTR+γ with the PHYML v 3.0 using automatic model selection [28]. Clustering was assessed by Cluster Picker software with the maximum genetic distances calculated by the program [31]. Monophyletic clusters were identified with Cluster-picker software using 3% within-cluster distance (substitutions/site) threshold and 0.9 aLRT bootstrap values. Phylogeographic interregional links within the clustered sequences were investigated.

Additionally, for the 321 clustered sequences Bayesian Monte Carlo Marcov Chain (MCMC) was used. Pooling of clustered sequences was performed for computational reasons. Three replicates of 35 million generations were run in BEAST v2.3.1 (2015) [32] using a coalescent constant population prior and a GTR model with uncorrelated relaxed exponential molecular clock [33]. This model was cosidered the most fit using phyML modeling. The molecular clock was calibrated using parameters derived from the phyML analysis, namely gamma shape: 0.33, estimated proportion of invariant sites with the initial value of 0.5, and the following GTR substitution rates: AC: 2.621, AG: 7.422, AT: 0.922, GC: 0.943, CT: 14.36, GT: 1.0. Lognormal priors were applied to the coalescent population size, proportion invariants sites for partitions and clock mean on partitions. All prior and posterior effective sample size (ESS) values exceeded 200. A consensus tree with posterior probabilities for branch support was obtained and annotated with TreeAnnotator v 1.5.4 using a 10% burnin. All trees were visualized in Figtree v.1.2.2.

Time between transmission events was calculated from the dated MCMC tree using distances between MRCAs for each cluster (internode intervals)—for each identified cluster/pair the time to the most recent common ancestor (tMRCA) was calculated. Medians and interquartile ranges for these intervals were calculated separately both for all clusters and clusters ≥5 sequences.

### Statistics

Statistical comparisons were performed using Fisher’s exact and Chi^2^ tests for nominal variables. Continuous variables were analysed using the Mann-Whitney *U*-test for nonparametric statistics (HIV viral load, age at diagnosis, baseline and nadir CD4 lymphocyte counts, internode intervals). Commercial software (Statistica 10PL, Statasoft, Warsaw, Poland) was used for above statistical calculations. Time trends for the frequency of transmission pairs and clusters were calculated using the logistic regression (R statistical platform version 3.1.0, package MASS). Two separate analyses (pairs + clusters ≥3 sequences and clusters ≥3 sequences only) were performed, with presence (coded as 1) or lack of clusters (coded as 0) investigated for every year. For analyses of time trends in the clustering the 707 (73.2% of the total cohort) patients diagnosed from 2009–2014 were included, as the sample size for the years 1989–2008 did not exceed 50 sequences. This sample (707 cases) represents 10.8% of the new HIV-1 diagnoses in Poland for the years 2009–2014 (6560 cases).

### Sequence data

Sequences from this study have been submitted to GenBank and may be accessed with the following IDs: KC409158-KC409222, KM057325-KM057372, KM283892-KM284680, KT340108- KT340205.

## Results

### Clustering among subtype B infections

**Fig 1:**
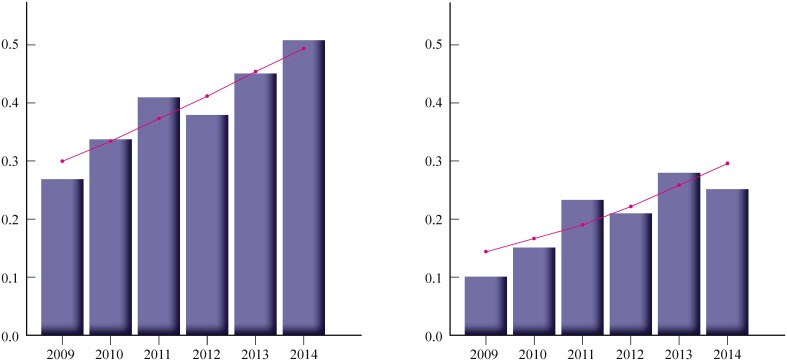
Proportions and logistic regression curves over time (2009–2014) for clusters/pairs (A, left) and clusters ≥3 sequences (B, right). Logistic regression curves are shown over the frequency bars.

Based on the constructed a maximum likelihood phylogenetic tree a total of 109 clusters and pairs containing 321 (33.2%) sequences were found, including 36 clusters of ≥3 individuals incorporating 165 (17.1%) sequences (S1 Fig). There were ten clusters of ≥5 sequences (n = 81, 8.4%), including two larger ones comprising 13 and 20 patients. All these clusters contained only individuals infected by the MSM route (shaded in grey at S1 Fig). Frequency of transmission pairs and clusters notably increased over time: from 26.92% in 2009 to 50.6% in 2014 [OR: 1.18 (95%CI: 1.06–1.31), p = 0.0026; slope for the frequency increase 2.8%/year]. This trend was similar when sequence pairs were excluded from the clustering analyses (increase from 9.62% in 2009 to 25.3% in 2014 [OR: 1.21 (95%CI: 1.06–1.38), p = 0.004; slope for the frequency increase 2.2%/year] (Fig 1).

### Clinical characteristics of clustered cases

Univariate associations between clustering and group characteristics are presented in Table 1. Sequence clusters/pairs were notably more frequent among MSM (229 cases, 71.3%) compared to other transmission routes (injection drug use (IDU): 32 (10.0%) and heterosexual transmissions: 60 (18.7%) cases, respectively [p<0.001, OR: 3.5 (95%CI: 2.6–4.6)]. Furthermore, clusters (groups of ≥ 3 cases) predominantly contained MSM-derived sequences (n = 131, 79.4%) with only 24 (14.5%) ones from patients infected by heterosexual contact and 10 (6.1%) by IDUs route. Moreover, patients with sequences forming pairs and clusters were younger at HIV diagnosis [median: 31 (IQR: 26–37) compared to 32 (27–39) years for unclustered sequences, p = 0.023], mostly asymptomatic at the time of analysis (67.8%, p<0.001), predominantly antiretroviral treatment naive (90.0%, p<0.001), and free of hepatitis C virus coinfection (83.3%, p<0.001). Additionally, CD4 lymphocyte counts at genotyping and CD4 lymphocyte nadirs were significantly higher for clustered/paired patients [baseline CD4 median: 407 (IQR: 209–565) cells/μl versus 290 (111–467) cells/μl, p<0.001 and nadir CD4 median 356 (IQR: 187–493) versus 223 (IQR: 88–383) cells/μl, p<0.001 for paired/clustered and unclustered sequences, respectively], while HIV viral load was similar across these categories. Those clinical, immunologic and virologic patterns were similar for clusters of ≥ 3 cases (after removal of sequence pairs from the analysis).

**Table 1:** Demographic, clinical and laboratory cohort characteristics associated with pairs and clusters ≥ 3 cases.

	Pairs and clusters ≥3 sequences	Unclustered	P value	Clusters ≥3 sequences	Unclustered	P value	Total
Male, n (%)	276 (85.98)	515 (79.84)	0.019	152 (92.12)	639 (80.78)	<0.001	791 (81.9)
Age at diagnosis, median years (IQR)	31 (26–37)	32 (27–39)	0.023	31 (26–36)	32 (26–39)	0.031	32 (26–39)
**CDC HIV infection stage at genotyping, n (%)**							
Stage A	215 (67.82)	295 (47.12)	<0.001	123 (75.93)	387 (49.55)	<0.001	510 (54.08)
Stage B	51 (16.09)	139 (22.2)		18 (11.11)	222 (28.43)		190 (20.15)
Stage C	51 (16.09)	192 (30.67)		21 (12.96)	172 (22.02)		243 (25.77)
**Dominant transmission route, n (% of the total)**							
IDU (intravenous drug use)	32 (9.97)	286 (28.68)	<0.001	10 (6.06)	207 (25.84)	<0.001	217 (22.5)
MSM (men having sex with men)	229 (71.34)	185 (44.34)		131 (79.39)	384 (47.94)		515 (53.3)
HET (heterosexual)	60 (18.69)	174 (26.98)		24 (14.55)	210 (26.22)		234 (24.2)
Lymphocyte CD4^+^ T cell counts at genotyping, median (IQR)	407 (209–565)	290 (111–467)	<0.001	411 (254–638)	307 (117–475)	<0.001	322 (126–503)
Nadir lymphocyte CD4^+^ T cell counts, median (IQR)	356 (187–493)	223 (88–383)	<0.001	354 (209–557)	238 (91–392)	<0.001	269 (104–410)
HIV viral load at genotyping, mean log copies/ml (SD)	4.80 (±1.0)	4.86 (±1.0)	0.71	4.80 (±0.8)	4.80 (±1.0)	0.68	4.80 (±1.0)
Antiretroviral treatment naive at HIV genotyping (%)	288 (90.0)	502 (77.83)	<0.001	155 (94.51)	635 (80.38)	<0.001	790 (81.87)
HCV coinfection	48 (16.67)	221 (37.52)	<0.001	17 (11.64)	252 (34.47%)	<0.001	608 (69.33)

CDC category available for 943 patients. Data for HCV coinfected for 877 patients.

In multivariate analyses of clusters/pairs adjusted for transmission route, gender, clinical category at diagnosis, antiretroviral treatment history (treated vs. naive) and HCV status the factors associated with clustering were MSM transmission route [OR: 2.24 (95%CI: 1.38–3.65), p<0.001] and CDC category A [OR: 1.93 (95%CI: 1.4–2.64), p<0.0001]. Similar results were obtained for clusters of ≥ 3 cases with OR: 2.093 (95%CI: 1.14–3.85), p = 0.018 for MSM transmissions and OR: 2.64 (95%CI: 1.71–4.04), p<0.0001 for asymptomatic infection at HIV diagnosis.

### Transmission networks

**Fig 2:**
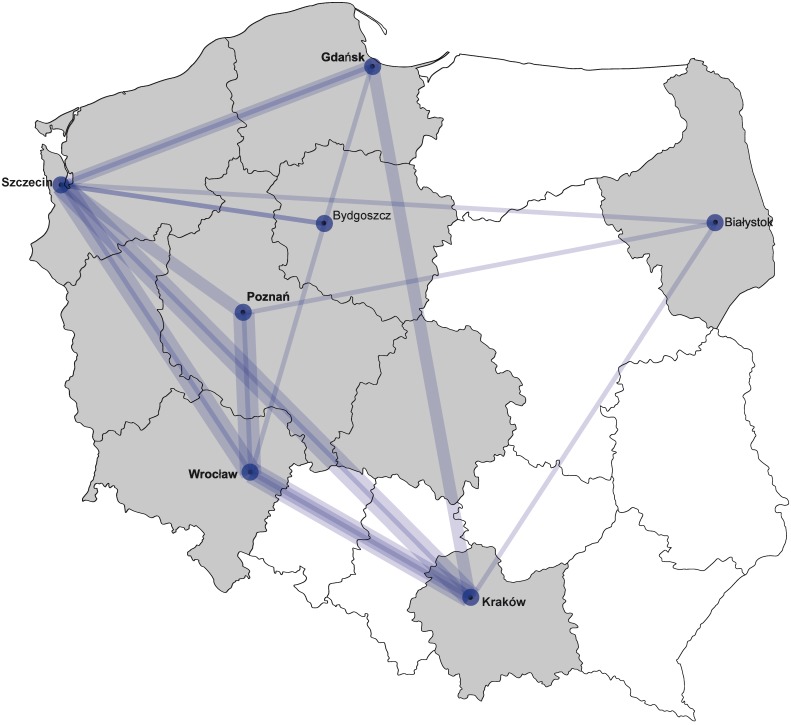
Interregional transmission networks identified in the Bayesian MCMC analysis. Line width and shading strength reflects the number of networks between sites. Provinces participating in the study are shaded in grey.

**Fig 3:**
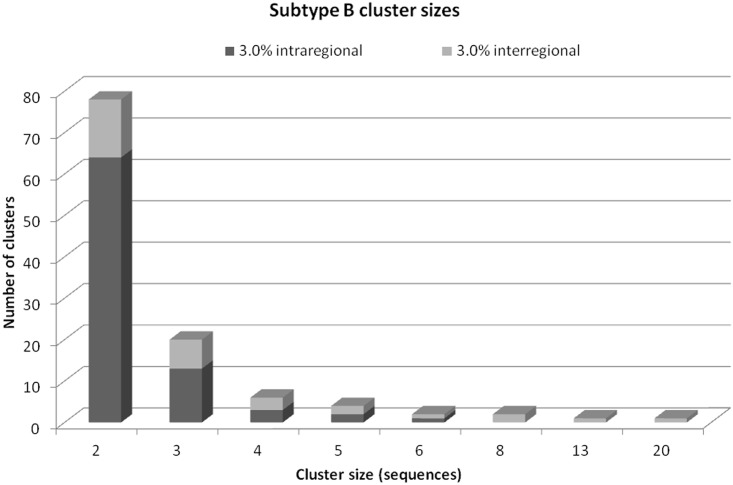
Cluster frequencies by size and type of clustering. Intraregional clusters are marked in dark grey, interregional in light grey.

To reflect the dynamics of the HIV transmissions in Poland in recent years we analysed the transmissions networks among the sites participating in the study. For this purpose the clusters and pairs containing sequences from patients followed-up at different centers were identified. Such interregional networks were found in 31 clusters/pairs containing 126 (13%) sequences from various centers (Fig 2) and were more common among MSM compared to other, transmission routes. In clusters/pairs containing patients from ≥2 centres 112 (11.6%) sequences were derived from MSM patients compared to four (0.4%) from IDU and 10 (1%) from heterosexually infected cases [p<0.001, OR:4.7 (95%CI: 2.55–8.96))]. As expected, frequency of clusters containing interregional sequences increased notably with the cluster size (OR for trend: 2.94 (95% CI: 1.51–5.64), p<0.001) (Fig 3).

### Time intervals between clustered transmission events

To further investigate phylogenetic relationships between the clusters and pairs dated Bayesian MCMC phylogeny using a subset of 321 closely related sequences forming clusters and pairs was reconstructed (marked in blue on the S1 Fig). Median genetic distances for clusters/pairs were 0.0199 (IQR: 0.013–0.0237) and 0.023 (IQR: 0.0199–0.0276) for clusters ≥ 3 sequences.

**Fig 4:**
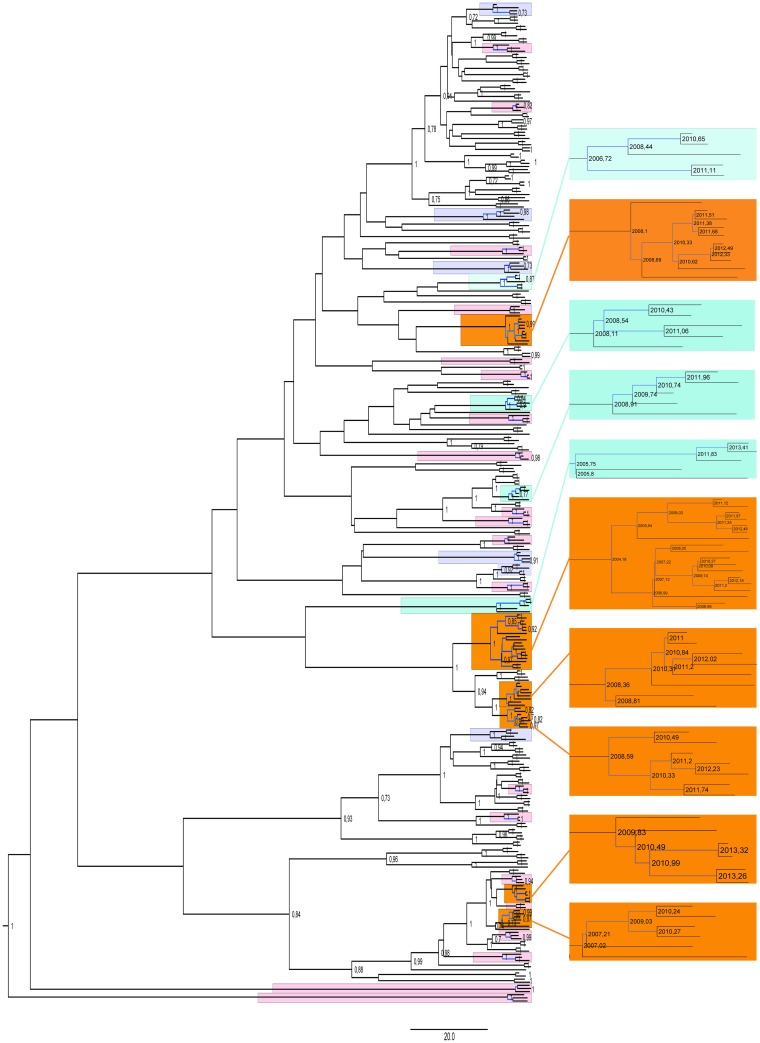
Bayesian, time-annotated MCMC phylogenetic tree of the subtype B *pol* sequences derived from clustered sequences coloured by cluster size. Clusters sized ≥5 have been enlarged. Branches used for the internode interval calculations are marked in blue. Posterior probabilities exceeding 0.7 are shown on the tree nodes.

To reflect the time between transmission events we have inferred a dated tree and calculated distances between tMRCA and previous nodes [20] for clusters of ≥ 3 sequences (Fig 4). Separate analysis was performed for clusters of ≥ 5 cases (exact topology for the time-scaled phylogenies for the clusters ≥ 5 shown in Fig 4). Time between the estimated times of infection for the clusters, reflecting time to onward transmission, ranged from 0.05 to 6.08 years [median 1.38 (IQR: 0.59–2.52) years]. For clusters of ≥5 sequences the internode intervals were significantly shorter compared to the smaller clusters [median 1.1 (IQR: 0.43–1.9) years versus median 2.13 (IQR: 1.1–3.66) years, p = 0.0026, S2 Fig]. Distribution of the internode distances for the clusters indicates that 22.4% of clustered onward transmissions occurred within the first 6 months, while 37.6% within the first year from infection; Analyzing only MSM clusters, the model suggests that the 23.1% and 35.9% of clustered transmissions occur within the first 6 months and one year from index case infection, respectively. This indicates that at least 3.9% (7.0% for MSM) of transmissions in the studied group occurred within 6 months and 6.6% (10.9% for MSM) within the first year of infection.

## Discussion

In the light of the rising number of new HIV diagnoses, clustered transmissions among MSM in Europe [4,34] and the shift in the character of epidemics in Poland from injection drug use related to concentrated, sexually-driven epidemics [6] we focused this study on the phylodynamic analysis of transmission networks. Data from different centers were analysed to minimize the sampling bias related to the localized epidemics and to reflect countrywide trends in the molecular epidemiology of HIV-1. The dataset included HIV sequences collected for genotyping purposes, and was limited to the subtype B as this clade remains the most prevalent in the country [11,12,35]. We have shown that the frequency (31%) of clustering is associated with MSM transmissions and early stage of HIV infection with common (13%) interregional transmission networks reflecting patient mobility Moreover, it was demonstrated that clustering is becoming more frequent over time, which indicates the uncontrolled, expanding epidemics and forward infections from individuals probably unaware of their HIV status, especially among MSM populations. This is in line with the surveillance data in Poland with the rapid increase in new HIV diagnoses in MSM, especially aged 24–34 years. In this group the number of newly diagnosed infections increased >20 fold, from 13 in the year 2000, to 313 in 2011 [6]. As of the most recent annual report (2013) 68.4% of newly diagnosed infections with disclosed transmission category were observed in MSM [7]. Clustering may only partially be explained by the earlier diagnosis during the course of infection over time, as the percentage of the late presenters reported from the epidemiological data was increasing [7].

Association between MSM transmission route and clustering within the subtype B infected cases has been previously reported in various cohorts [18,19,25,36,37,38]. Clinical characteristics of the clustered sequences followed the patterns observed in other studies including lack of prior antiretroviral treatment exposure, higher CD4 lymphocyte counts, earlier stage of HIV disease and more recent sampling [16,21]. Also observed clustering frequencies were within the 18.2–60% range observed across European and North-American datasets [16,36,37,39,40] and similar to the 31.2% observed in SPREAD cohort using the same internode genetic distance threshold of 3% [41]. Transmission networks observed in our study were not limited to the single geographical site with 13% of the total analyzed sequences (39.25% of the clustered ones) dispersed across HIV treatment centers. Of note, this may be associated with clustering networks, but also reflects the patient migration for personal and professional reasons.

It should be noted, that in the aforementioned SPREAD report, within a much smaller dataset of 193 Polish sequences from different geographical sites than the current study, a large cluster of 15 sequences was found. This is consistent with the identification of large, interregional clusters in our analysis including the ones comprising of 13 and 20 sequences. These large clusters may reflect rapidly growing sub-epidemics [19] and have been found throughout subtype B infected populations worldwide [38,42,43,44,45], however the exact transmission pattern within the transmission clusters is difficult to analyze in the cohort of chronically infected individuals. As expansion of the clusters over time has been reported [19,46] this finding supports the need for early introduction of antiretroviral therapy in populations with frequent clustering aiming to break the transmission chains [47].

Additionally, we have modeled the onward transmission time for the clustered infections indicating that 50% of clustered transmissions occur within 16.5 months after infection, with 25% within 7 months and 75% within 30 months.

Although interval modeling was performed using data inferred from samples with unknown duration of HIV infection, the results are highly consistent with the ones obtained for the Dutch observational cohort Athena [18] with reported median time to onward transmissions of 17 months (IQR: 7–17 months). Moreover, our data are in accordance to the study by Lewis et al., indicating that 22.4% (23.1% among MSM) of new infections occurred within the first 6 months from the index case [20]. Also, our observation that in the clusters ≥5 sequences 25% percent of transmissions occurred during the first 5.3 months and 50% of transmissions within the 13.2 months is consistent with the report by Brenner et al., modeling that 25%–30% of clustered transmissions occur within 6-months and 50% over a 14- to 17-month period [19].

These data, coupled with the temporal increase in the number of small and larger clusters, confirms the role of undiagnosed, recently infected individuals in fuelling of the current HIV epidemics in Poland, similarly to Canada, UK, Netherlands or France [18,19,25,48]. Our study is expanding the evidence on the enlarging MSM epidemics in Poland with the increasing importance of transmission networks and shows that current molecular dynamics of HIV spread reflects that of the West-European countries. One of the consequences of expanding transmission networks may be the increase in frequency of primary drug resistance [42,44], however, we have shown previously that trends in drug resistance were stable over time, with infrequent transmission within clusters and median prevalence of 8.3% [13,49]. Given the fact that estimated tMRCA infection times are within the timeframe of increased frequency of sexually-driven HIV infections observed over the last 10 years we suggest that clustered transmissions may significantly contribute to the current increase in the prevalence of HIV in Poland. These data are relevant for the design of prophylactic interventions and emphasize the need for prevention programs to focus on early detection and treatment among MSMs as it may reduce the frequency of onward transmissions within the networks [47,50]. Of note, prevention campaigns targeted to MSM in Poland are currently limited to anonymous counseling and testing, with no pre-exposure prophylaxis available. Scarce programs such as "Test and Keep in care"–program targeted at identification and referral to care for the newly diagnosed patients—do not seem sufficient as testing rates among populations at risk remain low [51]. The strategy should be "Seek, Test, Link, Treat, and Retain” as suggested by Hull at al. [52] and may be implemented across all populations, not only MSM.

Limitation of the study is related to the convenience of sampling—certain proportion of cases representing intermediate transmissions may have not been indentified and therefore not included in the analysis. This may result in the overestimation of the transmission intervals. Additionally, the data are limited only to the centers participating in the study—mostly Western and Southern provinces, however this is the largest sequence dataset available for the multiple sites in the country. Also, it should be emphasized that sequence datasets are not sufficient to determine the specific transmission events and ideally should be coupled with contact information [53]. It should also be noted, that definitions of genetic distance based clustering are inconsistent across the published studies ranging from 1.5% (most widely used for recent infections and local cohorts) to 4.5% (chronically infected individuals from nationwide analyses) [19,25,36,40,54]. We have used the 3% threshold for the intercluster genetic distances proposed by Kaye et al. [44] and used by the European Spread cohort [41].

## Conclusions

Clustering networks reflect expanding HIV epidemics among MSM in Poland. Interregional clustering reflects high dynamics of the nationwide HIV epidemics in the MSM group. Presented molecular data allow to emphasize the need for the evidence-based interventions which should focus on the proactive identification of populations at the highest risk, testing and early referral to antiretroviral treatment. Pre-exposure antiretroviral drug use should be implemented as preventive strategy among MSM in Poland as this group contributes significantly to onward HIV transmissions.

## Supporting information

S1 Fig Maximum likelihood tree showing the indentified clusters marked in blue. Clusters ≥ 5 sequences shaded in grey. All clustered sequences were used for the MCMC analysis using beast.
(PDF)

S2 Fig Histograms presenting distributions in time between the dates of infection for the clustered sequences for all (a) sequences/pairs, (b) clusters ≥ 5 sequences, (c) clusters of 2–4. (TIF)
