# Supplementary figures and images for: Expanding HIV-1 subtype B transmission networks among men who have sex with men in Poland
Source: PLoS One. 2017 Feb 24;12(2):e0172473. doi: 10.1371/journal.pone.0172473 (PMC5325290; doi:10.1371/journal.pone.0172473)

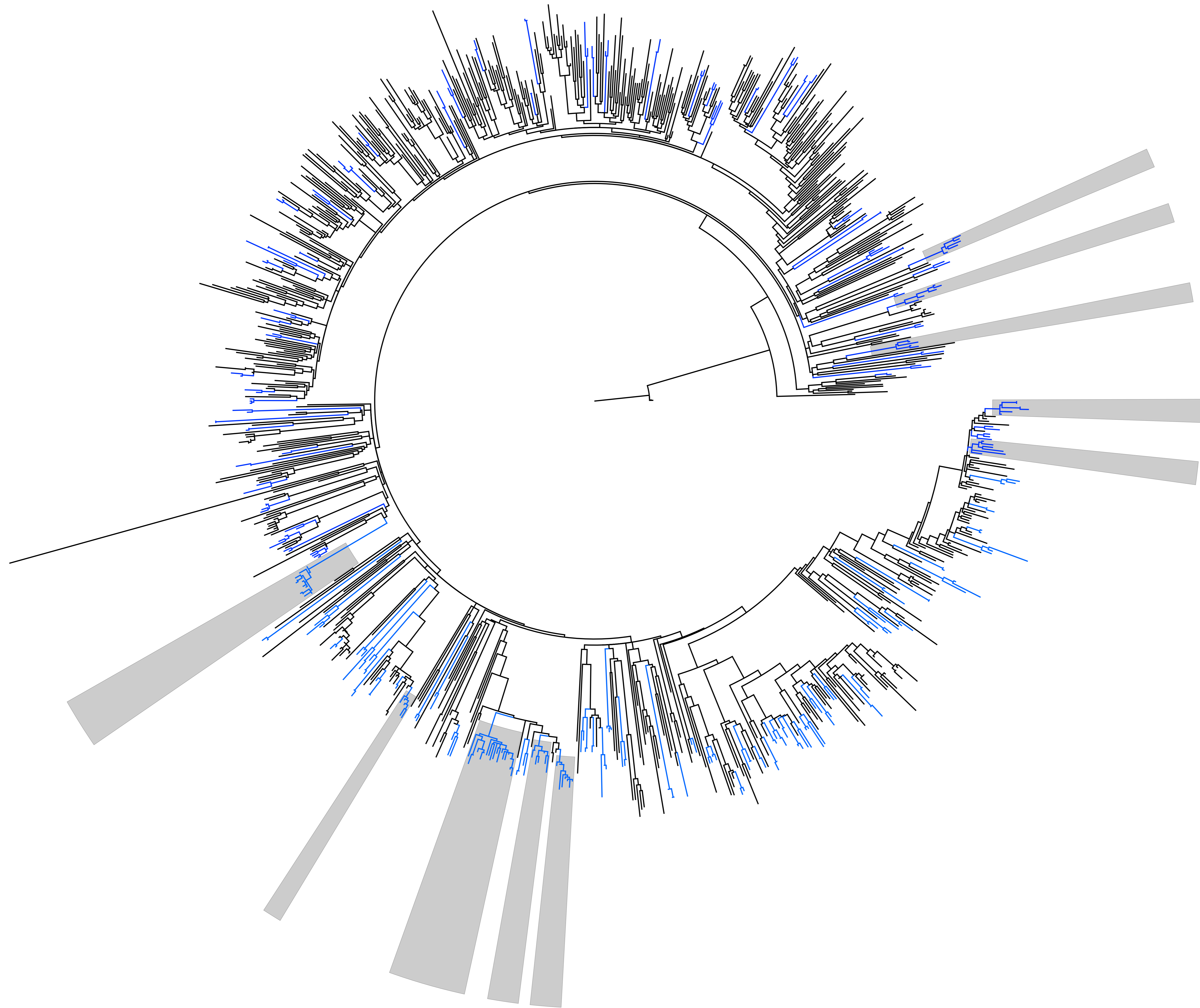

0.05

Supplement: S1 Fig — Clusters ≥ 5 sequences shaded in grey. All clustered sequences were used for the MCMC analysis using beast. (PDF) [file pone.0172473.s001.pdf]

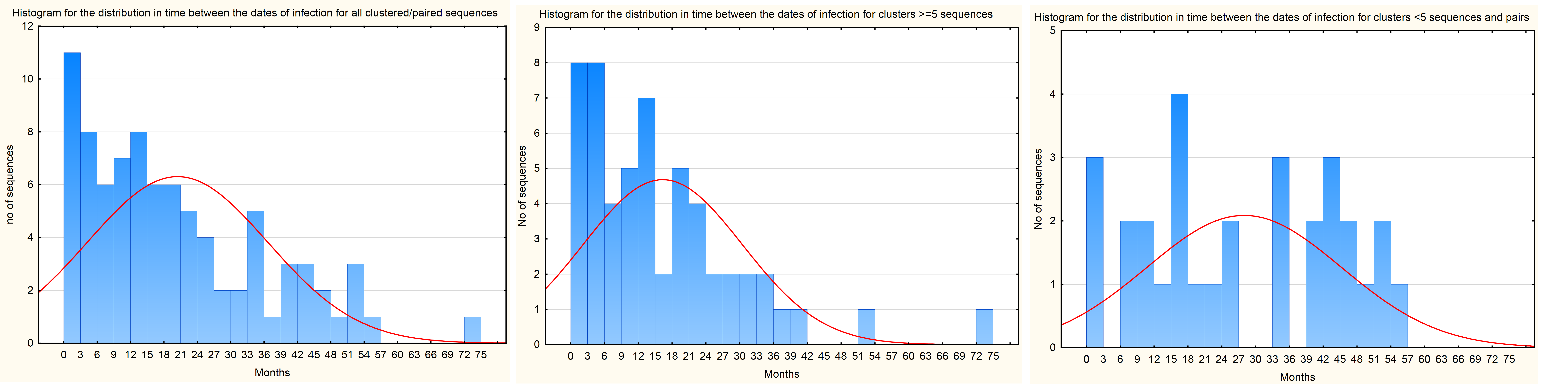

Supplement: S2 Fig — (TIF) [file pone.0172473.s002.tif]
